# #SexyBodyPositive: When Sexualization Does Not Undermine Young Women’s Body Image

**DOI:** 10.3390/ijerph20020991

**Published:** 2023-01-05

**Authors:** Daniela Di Michele, Francesca Guizzo, Natale Canale, Fabio Fasoli, Francesca Carotta, Arianna Pollini, Mara Cadinu

**Affiliations:** 1 Department of Developmental and Socialization Psychology, University of Padova, Via Venezia 8, 35131 Padova, Italy; 2 School of Psychology, Stag Hill Campus, University of Surrey, Guildford GU2 7HX, UK

**Keywords:** body positivity, beauty ideals, sexualization, Instagram, TikTok, body satisfaction, appearance social comparison, problematic social networking site use, body image, experimental study

## Abstract

Research suggests that exposure to social networking sites portraying a thin and often sexualized beauty ideal reduces young women’s body satisfaction, while exposure to body-positive content improves it. However, it is unclear whether sexualization could impair the beneficial effects of body-positivity messages. Young Italian women were exposed to one of three experimental conditions showing sexualized beauty ideals, sexualized body positivity, or non-sexualized body positivity that appeared either on Instagram (Study 1, *N* = 356) or TikTok (Study 2, *N* = 316). Across the two studies, results showed that, regardless of sexualization, exposure to body positivity increased body satisfaction and positive mood compared with pre-exposure measures, while exposure to sexualized beauty ideals reduced it. Participants in the sexualized beauty ideal condition also engaged in upward appearance social comparison whereas body positivity elicited downward comparison. Problematic social networking sites’ use moderated the effects of condition on body satisfaction, appearance social comparison, and positive mood, while downward comparison mediated the relation between condition and body satisfaction and positive mood. Our results highlight both beneficial and critical aspects of body positivity that should be taken into consideration when designing body image interventions and policymaking.

## 1. Introduction

Social networking sites (SNS) have become an essential part of our daily life. The number of people connected online is constantly increasing and visually-oriented social networks, such as Instagram and TikTok, are rising in popularity [1]. This brings attention to the negative consequences of such SNS use (e.g., [2]) but also to the potential that SNS can have in promoting well-being, for example by improving social connectedness [3] or promoting body-positivity movements [4].

SNS are particularly important in the context of women’s body image. Indeed, they play a role in representing women and promoting beauty ideals that increase female users’ body image disturbance (see [5,6] for reviews). Exposure to SNS images and videos depicting women conforming to cultural beauty standards (i.e., thin and fit, and often sexualized) has been associated with internalization of idealized beauty canons, self-objectification, appearance social comparison, and body dissatisfaction [7,8,9,10,11,12,13]. In response to such beauty ideals, in recent years, SNS have seen the rise of movements that seek to promote more inclusive and diverse beauty standards, such as *body positivity*. As a matter of fact, exposure to body-positive content has been found to improve women’s body satisfaction and appreciation [4,14]. However, several concerns about body positivity have been raised by scholars [4]. One of the criticisms concerns the fact that women in body-positive posts are often portrayed in a sexualized fashion (e.g., scantily dressed and in sensual poses), which may maintain the focus on women’s appearance rather than on other characteristics. A content analysis of body-positive Instagram profiles found that more than 32% of the content included elements of sexualization [15]. Moreover, Cohen and colleagues [14] showed that, while increasing body satisfaction, exposure to body positivity also increased young women’s self-objectification, in a similar way that exposure to beauty ideal content does. Likewise, Vendemia et al. [16] found that exposure to body positivity increased women’s self-objectification even when controlling for the degree of sexualization of the images.

Despite its relevance and calls for research on this topic [4], research on body positivity has posed very little attention to the issue of sexualization (see [16] for one exception). To our knowledge, research on the impact of sexualization in body-positivity posts on young women’s body image (e.g., body satisfaction) is not available. Hence, we investigated the effect of viewing sexualized vs. non-sexualized body-positive content on young women’s appearance social comparison, body satisfaction, mood, self-objectification, and intentions toward cosmetic surgery, compared to exposure to sexualized beauty ideal content. In doing so, we conducted two experimental studies that considered different social networking platforms, namely Instagram (Study 1) and TikTok (Study 2). We focused on Instagram and TikTok as they are both visually oriented social networks (i.e., they involve sharing of images and videos on Instagram and only videos on TikTok) and have a strong following among young people. Most 18–29-year-olds report using Instagram (71%) and TikTok (50%) [17], although TikTok might attract slightly younger users as 42% of them are below 25 years old compared to 30% on Instagram [18,19]. However, there are also differences between these two SNS. Instagram’s primary use is for posting and sharing still images [20]. Instead, TikTok only distributes videos of up to two minutes [21] that mostly involve music and moving content such as users dancing or doing challenges [22]. Thus, TikTok videos may attract users’ attention to appearance-focused content of moving bodies for a longer time and, perhaps, in more intense sessions in comparison to Instagram [23]. Importantly, despite its popularity among young women, TikTok has been rarely considered in the context of body image (but see [23,24]). Since Instagram has attracted more research, a comparison between the two platforms is particularly compelling.

### 1.1. Social Networking Sites: Sexualization, Beauty Ideals, and Body Image

According to the objectification theory [25], living in a context where women’s body is sexualized contributes to women’s sexual objectification, which occurs whenever women are treated as sexual objects, only useful to satisfy others through their physical appearance. It is well known that mass and social media play a decisive role in spreading a culture of sexual objectification, influencing perceived social norms and cultural beauty standards, through the dissemination of content that propose a stereotyped and sexualized representation of women [25,26,27]. While in the past decades traditional media (e.g., television, magazines) have played a key role in this trend [28], in more recent years, SNS have gained power, eventually overshadowing traditional media [1,29,30]. Experimental and cross-sectional studies suggest that the use of visually oriented social networks (e.g., Instagram, TikTok), compared to text-based ones (e.g., Facebook, Twitter), is more consistently related to dysfunctional body image, a stronger focus on physical appearance, higher self-objectification, and higher social comparison (see [31] for a review). These platforms allow users to show others an idealized and controlled representation of themselves as individuals edit and apply filters to their photos and videos, select the best shot, and choose lighting and poses that make them look their best [32,33]. In this way, these platforms offer a constant flow of images and videos that convey idealized and unattainable beauty standards enhancing women’s body concerns while making physical appearance a major focus point [34,35]. 

Along with the popularization of unrealistic beauty standards, visually oriented SNS contribute to maintaining a culture that sexually objectifies female bodies [27]. This is reflected in representations of the cultural ideal of female beauty that often include sexualization (i.e., provocative clothing and poses; see [36,37,38] for content analyses). Such ascendency of a single, narrow representation of femininity (i.e., hypersexuality) may result in a corresponding narrowing of culturally acceptable ways of expressing femininity [39], with consequent negative outcomes for both female children and women’s body images. In fact, experimental research on SNS has shown that exposure to idealized (and potentially sexualized) beauty imagery (often linked to “thinspiration” and “fitspiration”) leads to increased body dissatisfaction, negative mood, and self-objectification in women [14,24,32,40,41,42]. Interestingly, Guizzo et al. [10] have recently found that exposure to Instagram sexualized pictures increases young women’s body dissatisfaction compared to exposure to non-sexualized pictures of similarly attractive women, demonstrating that the impact of sexualization on women’s body dissatisfaction goes beyond the thin beauty ideal representations. Therefore, in line with objectification theory assumptions [25], sexualization in SNS may play a pivotal role in young women’s body image, especially when associated with bodies conforming to the cultural female beauty ideal (e.g., thin and fit). 

Considering the degree of sexualization and focus on beauty promoted by traditional and social media, it is not surprising that, over the past years, the desire to undergo cosmetic surgery has grown [43], especially among women [44]. Intense use of social media has been associated with a higher propensity of women to pursue cosmetic surgery [45], especially if they viewed content depicting people who have themselves undergone cosmetic surgery [46]. Moreover, both Instagram-image-based activities related to celebrities and exposure to social media influencers in TikTok predict greater cosmetic surgery intentions among young female users [47,48]. Overall, these findings highlight the role played by SNS in the development of body concerns and favorable attitudes toward cosmetic surgery as they might be a setting capable of convincing women that flawless, sexy bodies are the supposed normality.

### 1.2. The Body-positivity Online Movement

Alongside the hegemonic idealized and sexualized representations of women’s bodies on SNS, we have seen the rise of content that goes against the grain, such as body positivity. The body-positivity (#bopo) movement rejects the narrow and stereotyped ideal of beauty by promoting the acceptance of all bodies regardless of their shape, size, ethnicity, characteristics, and abilities [49]. Body positivity has found its expression on SNS [50] through the sharing of photos and videos that are not edited, posed, or filtered. It aims to enhance inclusion and celebrate the diversity of all bodies while promoting a positive body image. 

Research on the impact of SNS body-positive content on body image is in its infancy and requires further investigation [4]. There exists initial evidence that exposure to body positivity might be beneficial for women’s body image. Indeed, a study using an ecological momentary assessment protocol [51] and a quasi-experimental intensive longitudinal study [52] showed that women can experience positive effects after daily exposure to #bopo content. Hence, following body-positivity accounts could be a way to safeguard a positive body image among young adults. Furthermore, experimental studies showed that exposure to body-positive content on Instagram can lead to improved mood and body satisfaction in adult women [53] and in young women [14]. In both studies, women were exposed to body-positive, thin-ideal, or appearance-neutral Instagram images, and findings demonstrated that those exposed to body-positive content reported higher levels of body satisfaction, body appreciation, and positive mood compared to those exposed to thin-ideal or appearance-neutral content. However, Cohen et al. [14] found that acute exposure to body-positivity content led female participants to increased self-objectification as well, pointing to potential contradictory effects. 

Indeed, the body-positivity movement has been criticized by some scholars (see [4,16] for a discussion). For example, it continues to place emphasis on appearance, albeit with noble intentions, thus creating the idea and pressure that one must necessarily love one’s body [4]. Moreover, content analyses show that the very same images that convey messages of body positivity are not free from sexualization and objectification [15] or subtle elements belonging to mainstream beauty standards (e.g., portrayals of mainly Caucasian ethnicity, focus on appearance, sexually suggestive poses, thin praise, etc.; see [54]). Surprisingly, it also often features content related to fitspiration, which is frequently imbued with sexualization [38] and leads to negative mood and body dissatisfaction [52,55,56,57]. Thus, it cannot be ignored that, despite the evidence of its beneficial effects on women’s body image [52], body-positivity content carries a certain degree of contradiction and ambiguity, which might explain the heightened self-objectification reported by Cohen et al. [14]. In line with this reasoning, sexualized body-positive images have been found to increase women’s self-objectification compared to neutral images but not compared to non-sexualized body-positive images [16], a result suggesting that the focus on appearance in body-positive content might drive the effects. Despite the concerns raised about body positivity, experimental studies examining the impact of different types of body-positive content on women’s body image outcomes are still missing. Notably, no study has investigated the effects of sexualization in body-positive content on body satisfaction, as well as no study has explored the impact of viewing body-positivity content on intentions to resort to cosmetic surgery to improve one’s appearance. Therefore, we examined whether exposure to sexualized body-positive content would impair the expected positive effects of body positivity. 

### 1.3. Appearance Social Comparison and Social Networking Sites

According to social comparison theory [58], human beings have an inherent drive for self-evaluation, which may concern several domains, including physical appearance. When objective references are not available, people come to evaluate themselves by comparison with others (i.e., social comparison; [58]). As a result, people will experience pressure toward reducing discrepancies between their characteristics and others’ when such aspects are considered important for them and in their culture. Conventionally, scholars (e.g., [59]) refer to upward and downward comparisons when one perceives themself as superior or inferior, respectively, to a target individual in a particular domain. Likewise, lateral comparison occurs when one considers themself at a comparable level to a target individual. Literature on social comparison and body image has found that social comparisons may result in positive or negative outcomes depending on the direction of the process. Upward-directed appearance-based social comparison has been associated with body dissatisfaction in women [60,61], whereas downward social comparison has been linked with increased body satisfaction [62,63]. These findings are consistent with the tripartite influence model of body image [64], which suggests that women internalize unrealistic cultural appearance ideals disseminated through the media and engage in appearance social comparisons that ultimately lead to body dissatisfaction. Interestingly, the role of appearance social comparison has been advocated regarding the influence of SNS on female body image [12,33,38,65]. SNS enable individuals to be constantly connected with others, increasing the opportunities for appearance social comparisons [31], which most of the time for female users happen to be in the upward direction [66]. Importantly, previous experimental studies have demonstrated that state appearance social comparison, activated after exposition to social media content, mediates the effect of viewing idealized Instagram images and TikTok videos on young women’s reduced body satisfaction and mood [24,42]. Furthermore, correlational studies have found appearance social comparison to mediate the relationship between SNS usage and self-objectification [67]. However, to our knowledge, no study has yet investigated the role of upward and downward appearance social comparison while viewing body-positive content. Some experimental studies have found that engaging in downward social comparison while viewing media images of non-attractive women or plus-size models results in greater body satisfaction compared to viewing images of attractive women and thin models [62,63]. Nonetheless, body positivity on social media (i.e., Instagram, TikTok) covers more variegated portrayals of women’s bodies and the mechanisms underlying their positive impact on young women’s body image require specific investigation, a research gap addressed in the present research. 

### 1.4. Problematic Social Networking Sites Use 

A growing body of research has suggested that the relationship between SNS exposure and body image outcomes is moderated by individuals’ use of SNS. Specifically, research distinguishes between SNS use and problematic SNS use. Problematic Social Networking Sites Use (PSNSU) has been defined as an uncontrolled and maladaptive pattern of SNS use linked with high functional impairment and characterized by overlapping symptoms with addictive behaviors (e.g., loss of control, tolerance, and preoccupation; [68]). Heavy Instagram use predicts higher body dissatisfaction [69], body concerns [70], as well as a greater desire for cosmetic surgery [46]. Importantly, Instagram addiction proclivity moderates the relationship between appearance-related photo activity on Instagram and body dissatisfaction [71]. Moreover, Instagram addiction proclivity increases young women’s cosmetic surgery intentions especially when exposed to posts containing objectifying (vs. non-objectifying) characteristics (e.g., sexualized images or objectifying comments; [10]). Overall, these findings suggest that those who report heavy and problematic SNS use might be particularly affected by the idealized and sexualized content available on SNS as they might perceive SNS as a reliable source defining individuals’ value [10,71]. Accordingly, we investigated whether PSNSU might moderate the effects of our manipulation on young women’s body image. Since we are investigating different types of social media content, PSNSU could have different effects. For instance, it may enhance the negative impact of sexualized body ideal content on women’s body image (in line with [10]) and decrease the positive effects of body-positivity content. We will explore if this is the case.

### 1.5. Overview

We aimed at extending previous studies on body positivity [14,16] by assessing whether sexualization embedded in body-positive messages might have potentially counterproductive effects on women’s body image. To do so, across two experimental studies, we investigated the effects of exposure to Instagram images (Study 1) and TikTok videos (Study 2) portraying sexualized or non-sexualized women promoting body positivity, or sexualized women conforming to the beauty ideal on young women’s body satisfaction, mood, social comparison, self-objectification, and cosmetic surgery intentions. Building on previous literature [10,14], our studies addressed young women aged 18–35 since they are the main users of Instagram and TikTok [72] and are more vulnerable to sexualization in media [28]. Additionally, compared to Instagram, TikTok has been rarely considered in the context of body image (but see [23,24]), thus making a comparison between the two social network platforms particularly interesting. 

In line with previous research [10,14,62], we expected that, across both studies, exposure to SNS portraying non-sexualized body-positivity content would induce higher body satisfaction (Hp 1a), and improved mood (Hp 2a) in young female participants compared to the pre-manipulation measurement, whereas sexualized beauty ideal imagery would decrease body satisfaction (Hp 1b) and worsen the mood (Hp 2b) compared to pre-exposure levels. Higher body satisfaction (Hp 1c), mood (Hp 2c) and lower appearance comparison in the upward direction (Hp 3) would be expected in the non-sexualized body-positivity condition compared to the sexualized beauty ideal condition. Concerning self-objectification, previous findings disambiguating sexualization in body positivity effects did not include a sexualized beauty ideal condition [16], thus we only explored whether non-sexualized body positivity would decrease self-objectification compared to sexualized beauty ideals (in line with [27]). Moreover, building on literature showing that sexualization increases body concerns [10,28] we explored whether exposure to sexualized body-positive content would impair the expected positive effects. A similar explorative approach was used concerning cosmetic surgery intentions as no research is available on this variable in the context of body positivity.

A series of joint analyses of the two studies were performed to address additional research questions. First, we explored whether the effects were similar or different across the two studies that were run in parallel: this allowed us to explore if Instagram and TikTok elicited similar effects (RQ1). Second, we examined the moderating role of PSNSU (RQ2). Since we were investigating different types of social media content, we reasoned that PSNSU could have different effects. In line with Guizzo et al. [10] and Lee [71], we explored whether PSNSU would enhance the negative impact of sexualized beauty ideal content on women’s body satisfaction and cosmetic surgery. We also explored the potential impact of PSNSU on body-positivity effects as no study was available to make specific predictions. Finally, we explored the mediating role of social comparison direction (RQ3). Specifically, building on research showing that social comparison is a key process explaining social media impacts [61,67,71,73,74], we explored whether body-positivity conditions (vs. sexualized beauty ideal) might have an indirect effect on body satisfaction, mood, self-objectification, and cosmetic surgery via lower engagement in upward social comparison. The model also considered the potential moderating role of PSNSU. 

## 2. Study 1–Instagram

Study 1 aimed to replicate previous findings showing that body positivity has a positive impact on women’s body image [4,14]. In doing so, we will extend the literature by comparing sexualized and non-sexualized body-positivity content.

### 2.1. Method

#### 2.1.1. Participants

Three hundred and seventy-five participants completed the whole questionnaire. However, based on the inclusion criteria (see Procedure section for details), we excluded *n* = 4 participants who did not self-identify as women, *n* = 6 participants that were older than 35 years old, and *n* = 5 participants that failed the attention check. Moreover, *n* = 3 participants were eliminated as they were too slow in completing the survey suggesting they have not paid enough attention to our stimuli and measures (Lower cut-off = Mdn of the compilation time (761 s) / 3; upper cut-off = Mdn of the compilation time x 3). The final sample included 356 Italian young women (*M_age_* = 24.98; *SD_age_* = 3.69). As displayed in Table 1, our sample included mostly highly educated, heterosexual participants, with a good balance between students and workers. On average, participants reported using the smartphone 34% of their daily time on social network apps. Participants were balanced across conditions: sexualized beauty ideal condition: *n* = 114; sexualized body positivity: *n* = 123; non-sexualized body positivity: *n* = 119. Before the data collection, a priori power analyses (α = 0.05, 1 − β = 0.80) were performed based on the effect sizes (minimum η_p_^2^ = 0.03) reported in previous research in this area [10,14]. Based on these analyses, we aimed at recruiting a minimum of *N* = 340 participants. The sensitivity power analyses (α = 0.05, 1 − β = 0.80) computed on the available experimental sample (*N* = 356) indicated that the largest minimal detectable effect (MDE) was equal to *f* = 0.15, which fell in the small effect area [75].

#### 2.1.2. Experimental Design

We employed a simple-one factor between-subjects design with three levels (condition: non-sexualized body positivity vs. sexualized body positivity vs. sexualized beauty ideals). The within-subjects factor time (before vs. after) was considered for body dissatisfaction and mood as they were measured both before and after the manipulation. 

#### 2.1.3. Measures and Materials

The measures and materials used in the study are outlined below in the order in which they were presented to the participants.

##### Body Satisfaction and Mood

We assessed state body satisfaction and mood before and after exposure to conditions via the state Visual Analogue Scale (VAS; [76]). This measure was implemented in several studies assessing body satisfaction and mood and proved to be reliable and sensitive to small changes across time [14,77]. Participants were asked to indicate on a continuum from 0 (*Not at all*) to 100 (*Very much*) how much at that moment they felt satisfied with their physical appearance, and weight, as well as how much they felt depressed, anxious, angry, confident, and happy. Items were presented in randomized order. Considering the time of assessment (i.e., pre- and post-exposure to conditions), we created two body satisfaction indexes, two negative mood indexes, and two positive mood indexes (i.e., one pre- and one post-exposure to conditions). The indexes were obtained by calculating for each participant the overall mean of body satisfaction (i.e., two items; *r_pre_*(356) = 0.79, *p* < 0.001, *r_post_* (356) = 0.88, *p* < 0.001), negative mood (i.e., three items; *α_pre_* = 0.64, *α_post_* = 0.69), and positive mood (i.e., two items; *r_pre_*(356) = 0.49, *p* < 0.001, *r_post_* (356) = 0.53, *p* < 0.001). Higher scores indicated higher levels of body satisfaction, negative mood, and positive mood, respectively.

##### Experimental Manipulation

Participants were randomly assigned to one of the three experimental conditions. In each condition, they watched a video lasting 2 min and 40 s that included 10 Instagram static images portraying sexualized women conforming to the cultural beauty ideal (*sexualized beauty ideals*) vs. women promoting body-positivity content either in a sexualized (*sexualized body positivity*) or not sexualized way (*non*-*sexualized body positivity*). To enhance ecological validity, the stimuli were taken from public Instagram accounts and the transition between the stimuli in each video replicated the scrolling of images on the main Instagram page. Usernames, likes, and comments were obscured while maintaining Instagram’s interface. Each image was displayed for 15 s. We selected images of young women to match the mean age of our targeted participants. To disambiguate the role of sexualization in body positivity, the degree of sexualization of the stimuli was manipulated following previous work [10,78]. The images of the sexualized beauty ideal condition were drawn from the study by Guizzo et al. [10], while the stimuli of the body-positivity conditions were pretested (see Supplementary Materials). The sexualized beauty ideal images depicted women conforming to the thin and fit beauty ideal that were also portrayed in a sexualized fashion, that is in bikini and sensual poses (e.g., emphasizing body parts such as breasts and buttocks; [39,79]. The sexualized body-positivity condition portrayed women with bodies not conforming to the cultural beauty ideal (e.g., overweight, with a disability, etc.) in bikini and sensual poses. In the non-sexualized body-positivity condition, we used images of women representing culturally non-conforming bodies (e.g., overweight, with a disability) or focusing on certain body parts to normalize common imperfections (e.g., focusing on the head to show acneic skin, vitiligo, or alopecia) and with no sexualization involved (e.g., casual clothes, neutral standing poses).

##### Appearance Social Comparison

Social comparison was measured using the State Appearance Comparison Scale [59]. To assess the degree of appearance social comparison, participants were asked to indicate on a continuum ranging from 0 (*No comparison*) to 10 (*Constant comparison*) how much they compared their bodies to those depicted in the video previously watched. A second item was used to assess the direction of the comparison. Specifically, participants who answered the previous question with a value greater than zero indicated how they felt while comparing themselves on a 5-point Likert scale (1 = *much worse*, 3 = *same*, 5 = *much better*). We re-coded the values so that the index could range from –2 = *much worse*, 0 = *same*, and 2 = *much better*. Thus, negative scores indicated an upward comparison, whereas positive scores represented a downward comparison with 0 scores referring to lateral social comparison.

##### Self-Objectification

Self-objectification was measured via the Likert version of the Self-Objectification Questionnaire (LSOQ; [80]). Participants rated how important 10 body attributes were to their bodily self-concept on an 11-point Likert scale (1 = *Not at all important*, 11 = *Extremely important*). Five body attributes are related to appearance (e.g., weight, sex appeal) and the other five are related to body competence (e.g., physical coordination, health). A unique index of self-objectification was calculated by subtracting the mean of the competency items (*α* = 0.72) from the mean of the appearance items (*α* = 0.72). Higher scores indicated greater self-objectification and positive scores indicated greater importance given to appearance- over competence-related attributes. 

##### Intention to Undergo Cosmetic Surgery

Cosmetic surgery intentions were assessed with the Consider subscale of the Acceptance of Cosmetic Surgery Scale (ACSS, [81]; Italian version validated by Stefanile et al. [82]). The Consider subscale measures participants’ willingness to undergo cosmetic surgery in the future and interest in cosmetic procedures [82]. Answers were provided on a 7-point Likert scale (1 = *Completely disagree*, 4 = *Neither agree nor disagree*, 7 = *Completely agree*). Ratings were averaged (*α* = 0.93), so that the higher the score, the higher the intention to undergo cosmetic surgery in the future.

##### Problematic Social Networking Sites Use (PSNSU)

PSNSU was assessed with the Bergen Social Media Addiction Scale (BSMAS, [83]; Italian version validated by Monacis et al. [84]). The BSMAS contains six items that reflect the basic elements of addiction (salience, mood modification, tolerance, withdrawal, conflict, and relapse). Each item addresses experiences within 12 months and is answered on a 5-point Likert scale (1 = *Never or very rarely*; 5 = *Very often*). The sum of each item score was calculated for each participant (*α* = 0.78), so that higher scores indicated higher levels of social media addiction proclivity. BSMAS demonstrated good construct and convergent validity as well as reliability [84].

#### 2.1.4. Procedure

Data were collected remotely without the presence of the experimenters through the online platform Qualtrics International Inc from January to February 2022. Participants were recruited on a voluntary basis by the authors and dissertation students via posting ads on online platforms (e.g., Facebook, Instagram, WhatsApp, Telegram) using a snowball sampling method. The ads contained the link to access the study along with a message specifying the survey duration (15 min) and inclusion criteria (gender: woman; age: between 18 and 35 years). No compensation was given for participation in the study. To limit demand characteristics, participants were told that the study aimed to investigate short-term memory processes of media content, a cover story previously proved to be successful [10]. Participants’ written informed consent was asked at this point. Next, participants’ pre-exposure mood and body satisfaction levels were assessed. Afterward, participants were invited to carefully watch one of three randomly assigned videos (i.e., sexualized beauty ideal condition, sexualized body-positivity condition, non-sexualized body-positivity condition) and to answer a few questions investigating short-term memory processes of media content. To support the cover story and reduce suspicion of other measures, participants were told that the memory task would be preceded by interfering tasks (i.e., the dependent variables of the study). Thus, after the manipulation, we measured appearance social comparison, post-exposure mood and body satisfaction, self-objectification, and cosmetic surgery intentions. Thereafter, participants completed the memory task which included one attention-check item and two manipulation-check questions (see Supplementary Materials for results). Next, participants filled in the measure of PSNSU along with a measure of daily use of the smartphone with its apps and functions (e.g., WhatsApp, Social Networks such as Instagram and TikTok, video games). Additionally, since in previous literature feminist identification was found to be associated with positive body image [85], we measured participants’ levels of identification with the feminist movement. However, since in our study feminist identification was not associated with any of our outcomes, this variable will not be further considered. Finally, participants provided sociodemographic information, read a written debriefing about the true study aims, and were asked to confirm their consent to data use. All participants provided consent to data use, thus they were retained in the analyses. The study was approved by the ethical committee for Psychological Research of the University of Padova (research protocol n° 4387).

### 2.2. Results

#### 2.2.1. Data Analyses Plan

To test our predictions concerning body satisfaction as well as positive and negative mood, we conducted mixed ANOVAs on each outcome with condition (sexualized beauty ideal vs. sexualized body positivity vs. non-sexualized body positivity) as between-subjects factor and time of assessment (pre- vs. post-exposure) as the within-subjects factor. One-way ANOVAs with condition (sexualized beauty ideal vs. sexualized body positivity vs. non-sexualized body positivity) as a between-subjects factor were conducted on the degree of appearance social comparison, the direction of the comparison, self-objectification, and cosmetic surgery intentions. We reported only statistically significant main and interaction effects to facilitate the reading. Non-significant effects are available via the Open Science Framework (OSF) (at https://osf.io/jav7n/). Pairwise comparisons (with Bonferroni correction) are also reported. Descriptive statistics and zero-order correlations are reported in Table 2 and Table 3, respectively.

#### 2.2.2. Body Satisfaction

A significant Condition x Time interaction, *F*(2, 353) = 20.67, *p* < 0.001, η_p_^2^ = 0.10, emerged (see Table 2 for descriptive data). Pairwise comparisons (Bonferroni correction) showed that, compared to pre-exposure levels, participants’ body satisfaction decreased in the sexualized beauty ideal condition (*p* < 0.001), while it increased in the sexualized body-positivity condition (*p* = 0.010). No significant time effects emerged in the non-sexualized body-positivity condition (*p* = 0.287). 

We also examined differences across conditions at each time point. No differences between conditions were observed before exposure to manipulation (*ps* = 1.00). At post-exposure levels, participants reported higher body satisfaction in the sexualized body-positivity condition than in the sexualized beauty ideal condition (*p* = 0.026). Moreover, participants in the sexualized beauty ideal condition tended to show lower body satisfaction than in the non-sexualized body-positivity condition, although the difference was not significant (*p* = 0.060).

#### 2.2.3. Mood

A significant main effect of time was found on positive mood (*F*(1, 353) = 9.79, *p* = 0.002, η_p_^2^ = 0.03). Lower positive mood was reported after the manipulation (*M =* 44.29, *SD =* 1.27) compared to pre-exposure levels (*M =* 46.44, *SD =* 1.17). Importantly, we found a significant Condition x Time interaction, *F*(2, 357) = 4.33, *p* = 0.014, η_p_^2^ = 0.03 (see Table 2 for descriptive statistics). Compared to pre-exposure levels, participants’ positive mood significantly decreased after exposure to the sexualized beauty ideal condition (*p* < 0.001). No other significant time effects emerged (*ps* > 0.416) indicating that positive mood remained unchanged in the sexualized and non-sexualized body-positivity conditions. Moreover, no significant differences were observed when comparing conditions at pre- (*ps* = 1.00) and post-exposure levels (*ps* > 0.107). 

The same analysis on participants’ negative mood showed only a significant main effect of time (*F*(1, 353) = 43.92, *p* < 0.001, η_p_^2^ = 0.11). Regardless of the condition, participants’ negative mood decreased post-exposure (*M =* 30.44, *SD =* 1.23) compared to pre-exposure levels (*M =* 34.14, *SD =* 1.16).

#### 2.2.4. Appearance Social Comparison

We found a significant effect of condition, on participants’ degree of appearance social comparison, *F*(2, 353) = 15.55, *p* < 0.001, η_p_^2^ = 0.08. Participants in the sexualized beauty ideal condition engaged more in appearance social comparison than those in both body-positivity conditions (*ps* < 0.001). No difference between sexualized and non-sexualized body-positivity conditions was observed (*p* = 0.369, see Table 2). 

Moreover, we investigated the direction of such comparison across conditions among the participants who engaged in appearance social comparison (*n* = 289). A significant effect of condition, *F*(2, 287) = 160.99, *p* < 0.001, η_p_^2^ = 0.53, indicated that participants in the sexualized beauty ideal condition felt worse when comparing themselves to women in the video than participants in both body-positivity conditions (*ps* < 0.001). No difference between sexualized and non-sexualized body-positivity conditions was observed (*p* = 0.432, see Table 2). One sample *t*-tests against the zero (i.e., lateral social comparison) revealed that participants engaged in upward comparison in the sexualized beauty ideal condition (*t =* 13.27, *p* <.001), whereas in both sexualized and non-sexualized body-positivity conditions they significantly engaged in downward comparison (*ts* > 7.59, *ps* < 0.001).

#### 2.2.5. Self-objectification and Cosmetic Surgery Intentions

Participants’ self-objectification and cosmetic surgery intentions were not affected by condition, *Fs*(2, 353) < 0.34, *ps* > 0.714.

### 2.3. Discussion

Study 1 showed that, in line with Hp 1b and previous research [10], participants exposed to Instagram sexualized beauty ideal content reported lower body satisfaction compared to pre-exposure levels. Moreover, exposure to body positivity was beneficial but, unexpectedly, only when it involved sexualization. Indeed, exposure to sexualized body positivity induced higher body satisfaction relative to pre-exposure levels and compared to the sexualized beauty ideal condition. Contrary to Hp 1a, no difference was found between pre- and post-exposure to the non-sexualized body-positivity condition, although participants tended to report higher body satisfaction in this condition compared to the sexualized beauty ideal condition (partially supporting Hp 1c). These results might suggest that the positive impact of viewing bodies non-conforming to the cultural beauty ideal on Instagram is stronger when those bodies are proudly exhibited rather than covered by clothes, in line with the idea that all bodies are good bodies, can be exhibited, and can be sexy (see Instagram content related to #allbodiesaregoodbodies; #allbodiesaresexy). 

Concerning mood, in line with hypothesis Hp 2b, exposure to sexualized beauty ideal images decreased participants’ positive mood compared to pre-exposures levels. However, compared to pre-exposure levels, participants’ positive and negative mood was not affected by the exposure to non-sexualized body positivity (contrary to Hp 2a) and sexualized body positivity. Moreover, contrary to Hp 2c, no differences across conditions were found. These findings are only partially in line with Cohen et al. [14] who found women to report a better mood in the body-positivity condition than in the idealized body condition and align with other research reporting null findings on mood [10,77]. 

Regarding appearance social comparison, participants in the non-sexualized body-positivity condition (in line with Hp 3) and in the sexualized body-positivity condition reported lower appearance comparison and felt better consequently (i.e., downward comparison) relative to those in the sexualized beauty ideal condition. Specifically, compared to lateral appearance comparison, participants exposed to sexualized and non-sexualized body-positive imagery significantly engaged in downward appearance comparison, whereas those exposed to sexualized beauty ideal content reported significantly greater upward comparison. These findings corroborate and extend previous research showing that exposure to beauty ideals [86,87] is linked with higher social comparison in the upward direction and comparing oneself with plus-size models results in downward comparison [62]. 

No differences across conditions were found on self-objectification. These results extend Vendemia et al. [16] by showing that sexualized and idealized Instagram images impact young women’s self-objectification similarly to body-positive imagery, regardless of sexualization levels and speak to the idea that body positivity might potentially have unwanted negative effects Finally, exposure to conditions did not affect participants’ intentions to undergo cosmetic surgery, a result somehow in line with previous research by Guizzo et al. [10] who also found no direct effect of Instagram sexualized images. 

Overall, these results extend the emerging literature on body positivity, disambiguating for the first time the impact of sexualization in body-positive Instagram content on young women’s body image. Despite the well-known negative effects of exposure to sexually objectifying media on young women’s body image (see [28] for a review), in the context of body-positivity sexualization does not appear to impair the expected positive effects of body-positive messages on young women’s body image. 

## 3. Study 2–TikTok

Given the initial evidence with Instagram images provided by Study 1, we wondered whether similar results would be found after exposure to TikTok videos. TikTok is a video-based social media platform that has been growing in popularity among young people [17], reaching about 1,700 monthly active users worldwide in 2022 and becoming the third most used visually oriented SNS [88]. So far, most of the experimental studies investigating the impact of SNS on body image involved exposure to Instagram static images [31]. Only a few focused on TikTok [23,24] and they did not address body-positivity effects. Therefore, in Study 2, we aimed to fill this gap by replicating our first study with TikTok videos, where movements and changing facial expressions are in place. 

### 3.1. Method

#### 3.1.1. Participants

In Study 2, a sample of 346 participants filled out the whole questionnaire. Based on the inclusion criteria (see Study 1 procedure section), we excluded *n* = 12 participants who did not identify as women, *n* = 7 participants that were older than 35 years old, and *n* = 5 participants who failed the attention check. Finally, *n* = 6 participants were eliminated since they were too slow (below the lower cut-off identified as in Study 1) in completing the survey. Thus, the final sample included 316 Italian young women (*M_age_* = 23.87; *SD_age_* = 4.19). All participants provided consent to data use; thus, they were retained in the analyses. As shown in Table 1, most of the participants were heterosexual, students or working students, with a high school diploma or higher qualifications. Participants reported using the smartphone 27% of their daily time on social network apps on average. Participants were balanced across conditions: sexualized beauty ideal condition *n* = 106; sexualized body positivity *n* = 105; non-sexualized body positivity *n* = 105. As in Study 1, based on a priori power analyses, we aimed at recruiting a sample of *N* = 339 participants. Our final sample was slightly smaller than anticipated. However, the sensitivity power analyses (α = 0.05, 1 − β = 0.80) computed on the available experimental sample (*N* = 316) indicated that the largest minimal detectable effect (MDE) was equal to *f* = 0.16, which fell in the small effect area [75].

#### 3.1.2. Materials and Procedure

Materials and procedures used in Study 2 were identical to those employed in Study 1. The only difference concerned the stimuli presented in the three conditions. In Study 2, participants randomly watched one of three videos containing 10 reels retrieved from TikTok (1:50 min long). Depending on the condition, the video portrayed either sexualized women conforming to the cultural beauty ideal or women promoting body-positivity content in a sexualized way or body-positivity content in a non-sexualized way. As in the previous study, to enhance ecological validity, the stimuli were taken from public TikTok accounts and presented by maintaining the social network interface (frame, logo, features) while obscuring usernames, likes, and comments. The transition between the reels replicated the way contents are displayed on the main page of TikTok (i.e., scrolling). Each reel was displayed for about 10–15 s and was soundless. Similar to Study 1, in the sexualized beauty ideal condition, reels portrayed women conforming to the cultural beauty ideal (i.e., thin and toned) in a bikini while engaging in sexualized poses or dances (e.g., emphasizing their breasts, showing their buttocks and their tongues). The body-positivity conditions showed women with different types of bodies, not conforming to the cultural beauty ideal (e.g., overweight, with skin imperfections, disability, etc.) who were either sexualized or not sexualized. Specifically, the sexualized body-positivity condition depicted women wearing bikinis while engaging in sexualized poses or dances, as in the beauty ideal condition. In the non-sexualized body-positivity condition, women were clothed and engaged in neutral poses, showing certain body parts to normalize common imperfections. The women portrayed in the videos were young to match the average age of our targeted participants. As in Study 1, we manipulated sexualization and submitted the stimuli to a pretest (see Supplementary Materials for details). As in Study 1, we measured participants’ body satisfaction (*r_pre_*(316) = 0.71, *p* < 0.001, *r_post_* (316) = 0.83, *p* < 0.001), positive mood (*r_pre_*(316) = 0.47, *p* < 0.001, *r_post_* (316) = 0.57, *p* < 0.001), negative mood (pre-exposure: α = 0.66; post-exposure: α = 0.70), degree of social comparison, direction of social comparison, self-objectification (competencies: α = 0.71, appearance: α = 0.78), cosmetic surgery intentions (α = 0.94), and PSNSU (α = 0.80). Indexes were calculated as in Study 1.

### 3.2. Results

The same analytical approach as Study 1 was used. To increase readability, we reported only statistically significant effects. Non-significant effects are available via the Open Science Framework (OSF) (at https://osf.io/jav7n/) (accessed on 28 December 2022). Descriptive statistics and zero-order correlations are reported in Table 2 and Table 3, respectively. 

#### 3.2.1. Body Satisfaction

A significant Condition x Time interaction was found, *F*(2, 313) = 20.88, *p* < 0.001, η_p_^2^ = 0.12 (see Table 2). Compared to pre-exposure levels, participants’ body satisfaction decreased in the sexualized beauty ideal condition (*p* < 0.001), while it increased in both the sexualized and non-sexualized body-positivity conditions (*p* < 0.001 and *p* = 0.044, respectively). Additionally, comparing conditions at pre-exposure levels, no differences across conditions were observed (*ps* > 0.100). On the other hand, participants’ body satisfaction post-exposure to the sexualized beauty ideal condition was significantly lower compared to both sexualized and non-sexualized body-positivity conditions (*p* < 0.001 and *p* = 0.010, respectively). No difference between sexualized and non-sexualized body-positivity conditions emerged at post-exposure levels (*p* = 0.127).

#### 3.2.2. Mood

A significant Condition x Time interaction, *F*(2, 313) = 12.59, *p* < 0.001, η_p_^2^ = 0.07 was found on positive mood. Compared to pre-exposure levels, participants’ positive mood significantly decreased after exposure to the sexualized ideal condition (*p* < 0.001). No other significant time effects emerged for sexualized and non-sexualized body-positive conditions (*ps* > 0.168). Moreover, no significant differences between conditions were observed pre-exposure (*ps* = 1.00), whereas at post-exposure levels positive mood was significantly lower in the sexualized beauty ideal than in the sexualized body-positivity condition (*p* = 0.021). No other post-exposure significant differences between conditions emerged (*ps* > 0.107). 

Concerning participants’ negative mood, a significant main effect of time (*F*(1, 313) = 31.13, *p* < 0.001, η_p_^2^ = 0.09) was found. Regardless of condition, participants’ negative mood decreased post-exposure (*M =* 28.42, *SD =* 1.30) compared to pre-exposure levels (*M =* 31.98, *SD =* 1.26). This effect was qualified by a significant Condition x Time interaction, *F*(2, 313) = 5.60, *p* = 0.004, η_p_^2^ = 0.03 (see Table 2). Compared to pre-exposure levels, participants’ negative mood decreased after exposure to both body-positivity conditions (*ps* < 0.001), whereas no significant time effects were observed in the sexualized beauty ideal condition (*p* = 612). Finally, no significant differences between conditions were observed either pre- (*ps* = 1.00) or post-exposure to the condition (*ps* > 0.301).

#### 3.2.3. Appearance Social Comparison

We found a significant effect of condition on participants’ degree of appearance social comparison, *F*(2, 313) = 15.93, *p* < 0.001, η_p_^2^ = 0.09 (see Table 2). Participants in the sexualized beauty ideal condition reported more social comparison than those in the sexualized body-positivity condition (*p* < 0.001), whereas only a non-significant tendency emerged compared to the non-sexualized body-positivity condition (*p* = 0.079, see Table 2). Moreover, higher appearance comparison was reported in the non-sexualized body-positivity condition compared to the sexualized body-positivity condition (*p* = 0.003).

Furthermore, a significant effect of condition was found on the direction of the comparison (*n* = 245), *F*(2, 243) = 127.90, *p* < 0.001, η_p_^2^ = 0.51. Participants in the sexualized beauty ideal condition felt worse when comparing themselves to women in the video than participants in the body-positivity conditions (*ps* < 0.001). No difference between sexualized and non-sexualized body-positivity conditions was observed (*p* = 0.578, see Table 2). One sample *t*-tests against the zero (i.e., lateral social comparison) revealed that participants engaged in upward comparison in the sexualized beauty ideal condition (*t =* 10.96, *p* <.001), whereas they engaged in downward comparison in both sexualized and non-sexualized body-positivity conditions (*ts* > 7.12, *ps* < 0.001). 

#### 3.2.4. Self-Objectification and Intentions to Undergo Cosmetic Surgery

Participants’ self-objectification and intentions to undergo cosmetic surgery were not affected by condition, *Fs*(2, 313) < 0.59, *ps* > 0.553.

### 3.3. Discussion

When considering TikTok videos, participants reported lower body satisfaction after exposure to the sexualized beauty ideal condition (in line with Hp 1b), and greater body satisfaction after exposure to sexualized body-positive contents. In line with Hp 1a, also participants in the non-sexualized body-positivity condition reported increased body satisfaction compared to pre-exposure levels. This last effect was not found in Study 1, where static Instagram images were presented. It is possible that the type of stimuli matters. TikTok dynamic videos, allowing several poses and framings of the same female target, might be better suited to elicit beneficial effects on body satisfaction than static images when non-sexualized body-positive stimuli are concerned. Indeed, attention is usually greater when sexual stimuli are involved (see [89] for a meta-analysis) but also when processing dynamic vs. static images [90] In both studies, non-sexualized body-positive targets had a lower proportion of exposed body compared to their sexualized counterparts potentially making participants pay such stimuli less attention. However, in such an instance, moving clips might have triggered higher attention, and thus an impact on body satisfaction, than static images depicting similar content. 

Moreover, regardless of the sexualization, participants’ body satisfaction was higher after both body-positive conditions compared to the sexualized beauty ideal condition, supporting Hp 1c and further corroborating Study 1. These results significantly extend the sparse literature on TikTok effects. For example, Pryde and Prychard [24] have recently shown that fit inspiration reels on TikTok are not sufficient to increase female users’ body dissatisfaction. Our results, thus, suggest that sexualization (i.e., scant clothing and sensual poses) might be a key feature of the negative impact of idealized TikTok videos on young women’s body satisfaction (in line with [10] focusing on Instagram). Moreover, our results showed for the first time the potential beneficial effects of body-positive messages disseminated through the TikTok platform. Specifically, in the context of TikTok, body positivity seems to increase young women’s body satisfaction regardless of the degree of content sexualization. With regard to mood, in line with Study 1 and Hp 2b, exposure to sexualized beauty ideal videos was associated with decreased positive mood compared to pre-exposure levels, whereas exposure to both body-positivity conditions did not affect participants’ positive mood. Contrary to Study 1 findings, participants’ negative mood decreased after exposure to both non-sexualized body positivity (in line with Hp 2a) and sexualized body-positivity conditions but was not affected by the sexualized beauty ideal condition. Thus, it seems that sexualized beauty ideals in TikTok videos hinder positive moods whereas body positive messages reduce negative moods regardless of their sexualization. These findings complement and extend previous research (e.g., [24]) by highlighting the beneficial impact of body-positive messages and corroborating the negative impact of sexualization in combination with thin and tone ideals. 

Differently from Study 1, participants in the non-sexualized body-positivity condition reported levels of appearance social comparison similar to those of participants in the sexualized beauty ideal condition, which were higher compared to participants in the sexualized body-positivity condition. The interpretation of this effect is challenging as this is the first study focusing on body-positive TikTok videos. It might be that specific features of the body-positivity videos drove the effects, but the available data are insufficient for us to suggest an interpretation. Further research is needed. For instance, studies using eye tracker techniques and isolating specific characteristics of body positivity may help disentangle these effects. Indeed, our stimuli were purposely created to reflect a broad range of representations (e.g., based on body size, disability, and skin problems), but a specific type of stimuli may exert specific effects. Moreover, we did not measure participants’ chronic appearance social comparison which might have been different across conditions, a possibility to be addressed in future research. Nevertheless, participants in the body-positivity conditions, regardless of sexualization, engaged in downward social comparison, whereas those exposed to sexualized beauty ideal content reported higher appearance comparison in the upward direction. These findings support Hp 3 and Study 1 and extend Pryde and Prychard [24] who focused on the degree of social comparison. 

As in Study 1, participants’ self-objectification and intentions to undergo cosmetic surgery were not affected by the type of TikTok video, a result in line with Guizzo et al. [10] with Instagram images. Overall, this study represents a relevant advancement in the literature addressing visual SNS. Very few differences were noticed when looking at the results of the two studies involving Instagram and TikTok. Joint analyses were carried out to understand the stability of these results and directly assess such potential differences. 

## 4. Joint Exploratory Analyses

We addressed three research questions by conducting exploratory joint analyses on Study 1 and Study 2 altogether. This procedure allowed us to test the stability of the effects regardless of the social networking platforms (Instagram vs. TikTok; RQ1) and explore moderating (RQ2) and mediating effects (RQ3) by relying on higher statistical power. Participants’ samples and the experimental design were very similar in Study 1 and Study 2, and the two data collections were conducted parallelly. Therefore, the statistical cross-examination of the studies is theoretically reliable (for a similar procedure, see [91,92]). The overall sample was *N* = 672. 

### 4.1. Instagram and TikTok Differences

The first research question (RQ1) concerned potential differences in the effects of our manipulation due to the type of social media network. To assess this, we conducted the same analyses as above including the study (Study 1-Instagram = 0 vs. Study 2-TikTok = 1) as a between-participants factor. Table 4 reports the descriptive statistics for all variables. 

Corroborating Hp 1a, 1b, 1c, results on body satisfaction showed the same pattern described in Study 2 (TikTok; Time x Condition *F*(2, 666) = 41.83, *p* < 0.001, η_p_^2^ = 0.11). No significant main effect of the study (*F =* 3.38, *p* = 0.067) or interactions of study with condition or time (*Fs <* 1.69, *ps* > 0.194) emerged. 

Concerning positive mood, the results were the same as Study 2 (Time*: F* (1, 666) = 11.72, *p* =.001, η_p_^2^ = 0.02; Time x Condition *F*(2, 666) = 15.79, *p* < 0.001, η_p_^2^ = 0.04) with the addition that lower positive mood was reported in the sexualized beauty ideal condition also compared to the non-sexualized body-positivity condition. The main effect of study was significant (*F*(1, 666) = 4.76, *p* =.03, η_p_^2^ = 0.01) but it did not significantly interact with condition or time (*Fs <* 1.09, *ps* > 0.335). 

Results concerning negative mood showed the same pattern as Study 2 (Time*: F* (1, 666) = 73.98, *p* <.001, η_p_^2^ = 0.10; Time x Condition *F*(2, 666) = 5.84, *p* = 0.003, η_p_^2^ = 0.01), with the addition that negative mood decreased in the sexualized beauty ideal condition compared to pre-exposure levels (*p =* 0.030). The main effect of study was not significant (*F =* 1.51, *p* = 0.219), nor it significantly interacted with condition or time (*Fs <* 1.85, *ps* > 0.159).

Moving to appearance social comparison, a significant condition main effect (*F*(2, 666) = 25.55, *p* < 0.001, η_p_^2^ = 0.07) showed an identical pattern of results to Study 1 (Instagram). However, a significant Condition x Study interaction emerged (*F*(2, 666) = 6.34, *p* = 0.002, η_p_^2^ = 0.02) indicating that Instagram participants reported lower social comparison in the non-sexualized body-positivity condition compared to TikTok participants in the same condition (see Table 2 for separate studies’ descriptive statistics). Condition effects on the appearance comparison direction were the same as in the two studies separately (*F*(2, 666) = 286.11, *p* < 0.001, η_p_^2^ = 0.52), and no effect of study was found (*Fs <* 0.03, *ps* > 0.950). 

Finally, a significant main effect of study was found on both self-objectification (*F*(1, 666) = 7.65, *p* = 0.006, η_p_^2^ = 0.01) and cosmetic surgery intentions (*F*(1, 666) = 18.96, *p* < 0.001, η_p_^2^ = 0.03). Instagram participants reported higher levels of both outcomes (self-objectification *M* = -.59, *SD* = 1.90; cosmetic surgery intentions *M* = 4.42, *SD* = 1.71) compared to TikTok participants (self-objectification *M* = -.99, *SD* = 1.88; cosmetic surgery intentions *M* = 3.83, *SD* = 1.78); no other significant results were found (*Fs <* 0.90, *ps* > 0.407).

Overall, only a few differences due to the type of study/stimuli emerged in relation to positive mood, self-objectification, cosmetic surgery intentions, and the overall degree of appearance social comparison. The latter variable was the only one where a significant interaction between study and condition occurred. Because of this result, the degree of appearance comparison was excluded from the analyses reported below, and study was included as a controlling variable. 

### 4.2. Problematic Social Networking Sites Use Moderation Effects

To test the moderating role of Problematic Social Networking Sites Use (PSNSU), we conducted separate multiple linear regression on body satisfaction, positive mood, negative mood, direction of appearance social comparison, self-objectification, and cosmetic surgery. We used PROCESS (Model n.1, [93]), with condition (sexualized beauty ideal vs. sexualized body positivity vs. non-sexualized body positivity), PSNSU and their two-way interactions as predictors. Condition was dummy coded using the indicator technique that took the sexualized beauty ideal condition as reference (=0), so that the first dummy X1 tested the effects of the sexualized body-positivity condition (=1) and the second dummy X2 tested the effects of the non-sexualized body-positivity condition (=1). PSNSU was mean-centered when interacting with the dummy variables. Given that study was a main predictor of some of the outcomes taken into consideration, we included it as a covariate (Study 1-Instagram = 0 vs. Study 2-TikTok = 1). Moreover, pre-exposure levels of body satisfaction, positive mood, and negative mood were included in the models as separate covariates (continuous) when appropriate. No multicollinearity issues were found (VIFs < 1.12). Huber-White (HC0) heteroskedasticity-consistent standard error estimator was used to correct for homoskedasticity violations [94].

**Figure 1:**
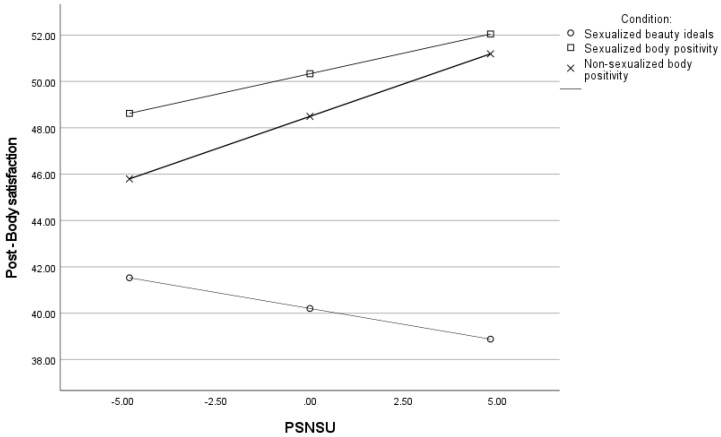
PSNSU moderating effect on the relation between condition and post-exposure body satisfaction.

Concerning body satisfaction, the overall model was significant. As shown in Table 5, we found a significant interaction between PSNSU and both X1 and X2: the higher participants’ PSNSU the higher their body satisfaction in both non-sexualized (*b =* 0.56, *t* = 2.14, *p* = 0.032) and sexualized body-positivity conditions (*b =* 0.35, *t* = 2.02, *p* = 0.044) compared to the sexualized beauty ideal condition, which showed a reversed, although not significant, pattern of results (*b =* −0.27, *t* = 1.62, *p* = 0.105) (see Figure 1). Please notice that the same results were found when not controlling for pre-exposure body satisfaction, but the model explained a significantly lower amount of variance, *F*(6, 665) = 12.77, *p* < 0.001, *R*^2^ = 0.08.). 

**Figure 2:**
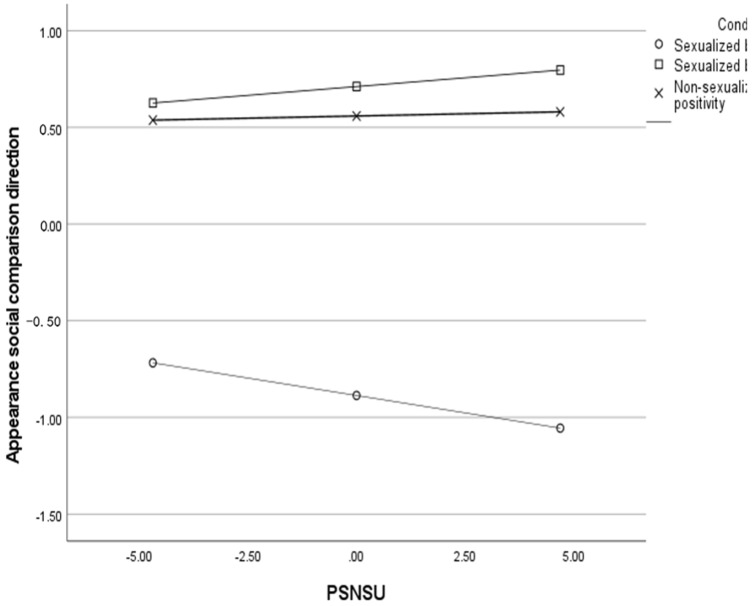
PSNSU moderating effect on the relation between condition and the direction of appearance social comparison.

Concerning the direction of appearance social comparison, as shown in Table 6, the higher participants’ PSNSU the worse they felt while engaging in social comparison (i.e., the more they engaged in upward comparison). Importantly, PSNSU significantly interacted with both X1 and X2, so that the higher PSNSU, the worst participants’ felt in the sexualized beauty ideal condition (*b =* −0.04, SE = 0.01, *t* = 2.82, *p* = 0.002) compared to both the non-sexualized (*b =* 0.004, SE = 0.01, *t* = 0.38, *p* = 0.701) and the sexualized body-positivity conditions (*b =* 0.02, SE = 0.01, *t* = 1.52, *p* = 0.129) (see Figure 2). 

As regards other outcomes, PSNSU positively predicted both self-objectification (*b = 0*.09, SE = 0.03, *t* = 3.62, *p* < 0.001; overall model *F*(6, 665) = 6.21, *p* < 0.001, *R*^2^ = 0.06) and cosmetic surgery intentions (*b = 0*.11, SE = 0.02, *t* = 5.28, *p* < 0.001; overall model *F*(6, 665) = 10.61, *p* < 0.001, *R*^2^ = 0.08), so that the higher the PSNSU, the higher participants’ self-objectification and cosmetic surgery intentions. Moreover, a significant interaction between PSNSU and X1 on positive mood was found but it did not significantly increase the amount of variance explained (ΔR^2^ = 0.003, *F* (2, 664) = 2.81, *p* = 0.061) so it will not be further discussed (see supplemental materials for complete analyses). No other significant results related to PSNSU emerged. 

### 4.3. Mediation Analyses

Building on research indicating appearance social comparison and, specifically, the direction of the comparison as a mechanism underlying SNS impact on body image concerns [24,42,62,63,74] and based on the moderation results above we ran moderated mediation analyses using PROCESS (model n° 8, [93]). Specifically, we entered condition (sexualized beauty ideal vs. sexualized body positivity vs. non-sexualized body positivity) as the independent variable, appearance social comparison direction (continuous) as the mediator, and body satisfaction, positive mood, negative mood, self-objectification, and cosmetic surgery as separate dependent variables. PSNSU was entered as the moderator on the relation between condition and both the mediator and the dependent variable. The dummy coding variables X1 and X2 were calculated exactly as the moderation analyses reported above. We included Study as a covariate (Study 1-Instagram = 0 vs. Study 2-TikTok = 1) as well as pre-exposure levels of body satisfaction, positive mood, and negative mood when appropriate. Huber-White (HC0) heteroskedasticity-consistent standard error estimator was used to correct for homoskedasticity violations [94]. Boot-strapped confidence intervals based on 5000 samples were computed to assess indirect effects. 

**Figure 3:**
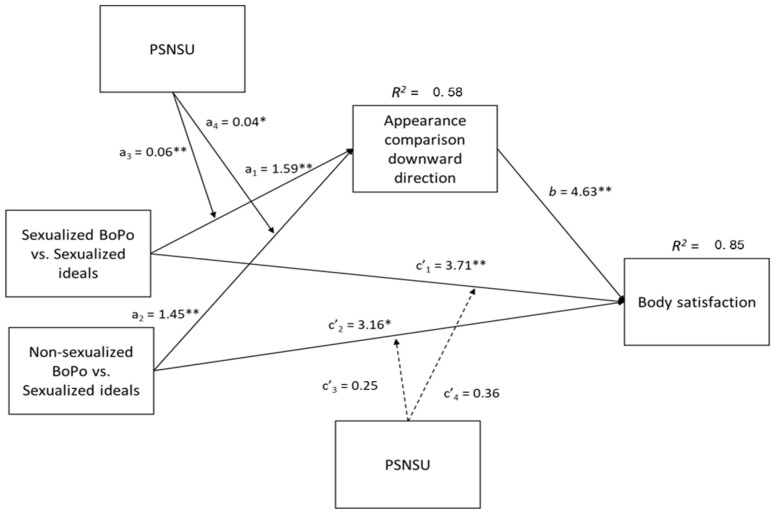
Moderated mediation model on post-exposure body satisfaction. Note: * *p* < 0.05; ** *p* < 0.001; Sexualized BoPo = sexualized body-positivity condition; Sexualized ideals = sexualized beauty ideal condition; Non-sexualized BoPo = non-sexualized body-positivity condition; PSNSU = problematic social networking site use; a_1_ = sexualized BoPo vs. sexualized ideals on downward appearance comparison; a_2_ = non-sexualized BoPo vs. sexualized ideals on downward appearance comparison; a_3_ = interaction PSNSU × X1 (sexualized BoPo vs. sexualized ideals) on downward appearance comparison; a_4_ = interaction PSNSU × X2 (non-sexualized BoPo vs. sexualized ideals) on downward appearance comparison; c’_1_ and c’_2_ = direct effects on body satisfaction; c’_3_ = interaction PSNSU × X2 (sexualized BoPo vs. sexualized ideals) on body satisfaction; c’_4_ = interaction PSNSU × X1 (non-sexualized BoPo vs. sexualized ideals) on body satisfaction.

Results on appearance social comparison mimicked exactly the moderation analyses above (see Table 6) and both body-positivity conditions predicted higher downward social comparison than the sexualized beauty ideal condition the higher the participants’ PSNSU (see Figure 3). The only difference was that the slope in the sexualized body-positivity condition was significantly different from zero (*b* = 0.03, SE = 0.01, *t* = 2.55, *p* = 0.011) so that the higher the PSNSU the better participants felt (i.e., the higher the downward comparison) in the sexualized body-positivity condition. Overall model, *F*(7, 528) = 128.42, *p* < 0.001, *R*^2^ = 0.58. In turn, higher downward social comparison predicted higher body satisfaction after manipulation exposure. The direct effects of both body-positivity conditions (vs. sexualized beauty ideal condition) on body satisfaction were significant, but PSNSU did not moderate the effects. Overall model, *F*(8, 527) = 554.20, *p* < 0.001, *R*^2^ = 0.85. The indexes of moderated mediation for both sexualized body positivity (vs. sexualized beauty ideal) (ω = 0.27, *boot SE* = 0.09, *CI* [0.10, 0.46]) and non-sexualized body positivity (vs. sexualized beauty ideal) (ω = 0.17, *boot SE* = 0.08, *CI* [0.02, 0.35]) were significant as the confidence intervals did not include the zero. Overall, both body-positivity conditions increased participants’ downward comparison compared to the sexualized beauty ideal condition and this effect was bigger the higher participants’ PSNSU. In turn, such downward comparison increased participants’ body satisfaction.

Please notice that the indirect effects of both body-positivity conditions (vs. sexualized beauty ideal condition) on body satisfaction via downward social comparison emerged at every level of PSNSU, but the effect was bigger the higher participants’ PSNSU. The indirect effects values for the sexualized body-positivity (vs. sexualized beauty ideal) condition were: −1 *SD* PSNSU *ab* = 6.09, *CI* [3.96, 8.57]; PSNSU mean *ab* = 7.38, *CI* [4.98, 9.93]; +1 *SD* PSNSU *ab* = 8.66, *CI* [5.81, 11.65]. The indirect effects values for the non-sexualized body positivity (vs. sexualized beauty ideal) condition are: −1 *SD* PSNSU *ab* = 5.91, *CI* [3.88, 8.22]; PSNSU mean *ab* = 6.71, *CI* [4.53, 9.05]; +1 *SD* PSNSU *ab* = 7.50, *CI* [5.02, 10.16].). 

**Figure 4:**
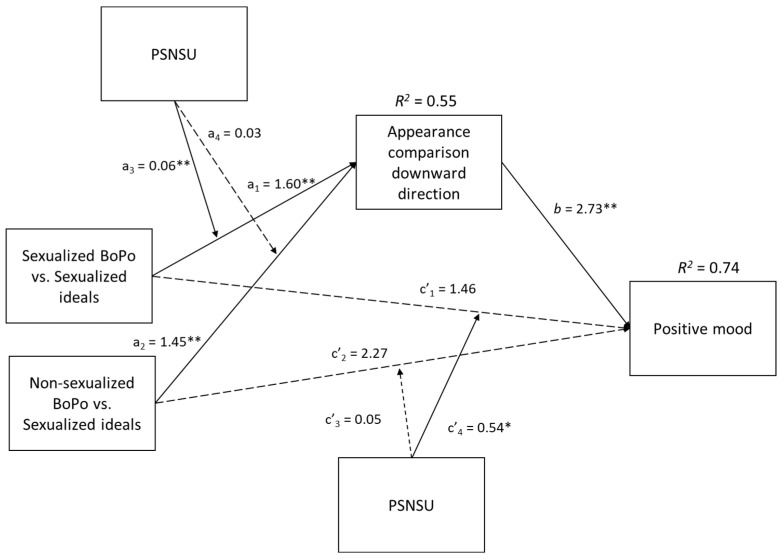
Moderated mediation model on post-exposure positive mood. Note: * *p* < 0.05; ** *p* < 0.001; Sexualized BoPo = sexualized body-positivity condition; Sexualized ideals = sexualized beauty ideal condition; Non-sexualized BoPo = non-sexualized body-positivity condition; PSNSU = problematic social networking site use; a_1_ = sexualized BoPo vs. sexualized ideals on downward appearance comparison; a_2_ = non-sexualized BoPo vs. sexualized ideals on downward appearance comparison; a_3_ = interaction PSNSU x X1 (sexualized BoPo vs. sexualized ideals) on downward appearance comparison; a_4_ = interaction PSNSU × X2 (non-sexualized BoPo vs. sexualized ideals) on downward appearance comparison; c’_1_ and c’_2_ = direct effects on positive mood; c’_3_ = interaction PSNSU × X2 (sexualized BoPo vs. sexualized ideals) on positive mood; c’_4_ = interaction PSNSU × X1 (non-sexualized BoPo vs. sexualized ideals) on positive mood.

A very similar pattern of results emerged on positive mood. Higher downward comparison driven by exposure to sexualized body positivity (vs. sexualized beauty ideal) and especially a higher PSNSU was linked with higher positive mood (see Figure 4). Overall model, *F*(8, 527) = 220.74, *p* < 0.001, *R*^2^ = 0.74. The moderated mediation index related to X1 was significant (ω = 0.16, *boot SE* = 0.07, *CI* [0.04, 0.31]) confirming the indirect effects of sexualized body-positivity (vs. sexualized beauty ideal) posts on positive mood via higher downward comparison and suggesting that this effect increased the higher was participants’ PSNSU (−1 *SD* PSNSU *ab* = 3.62, *CI* [1.46, 5.96]; PSNSU mean *ab* = 4.37, *CI* [1.78, 7.03]; +1 *SD* PSNSU *ab* = 5.12, *CI* [2.05, 8.26].). On the contrary, X2′s moderated mediation index was not significant (ω = 0.08, *boot SE* = 0.05, *CI*[−0.004, 0.21]. Specifically, the indirect effect of the non-sexualized body-positivity (vs. sexualized beauty ideal) condition on positive mood via downward social comparison emerged at every level of PSNSU without significant differences the higher it incremented (−1 *SD* PSNSU indirect effect = 3.56, *CI* [1.41, 5.87]; PSNSU mean indirect effect = 3.97, *CI* [1.60, 6.44]; +1 *SD* PSNSU indirect effect = 4.38, *CI* [1.78, 7.13]). 

No other significant moderated mediation effects emerged on the other outcome variables. 

## 5. General Discussion

Literature has provided initial evidence of the beneficial effects of body-positive messages on young women’s body image [14]. Nonetheless, posts promoting body positivity on social media frequently involve portrayals of sexualized women [15]. Surprisingly, the impact of sexualization on body-positivity SNS messages has not received much attention (but see [16] for an exception). To address this gap, across two studies focusing on Instagram and TikTok, we investigated the impact of exposure to sexualized vs. non-sexualized body-positivity images/reels on young women’s body satisfaction, mood, appearance social comparison, self-objectification, and intentions to undergo cosmetic surgery, relative to exposure to sexualized beauty ideal content. 

Across platforms and medium (static images vs. videos), sexualization did not compromise the expected benefits of body-positivity messages. In fact, exposure to body-positive images/reels, regardless of sexualization, increased body satisfaction (partially in line with Hp 1a). Conversely, we confirmed and extended previous research [10] by showing that exposure to sexualized beauty ideal content reduces participants’ body satisfaction (Hp 1b) and positive mood (Hp 2b). Additionally, body satisfaction was lower when posts involved sexualized beauty ideals rather than both non-sexualized (Hp 1c) and sexualized body-positivity posts). Therefore, sexualization appears to work differently depending on the type of content to which women are exposed, resulting in body dissatisfaction and lower positive mood only when combined with representations of the cultural beauty ideal. One might conclude that sexualization can be beneficial within the body-positive movement, as bodies non-conforming to the cultural beauty ideal proudly exhibited rather than covered by clothes might help support the message that all bodies are good, can be exhibited, and be sexy (#allbodiesaregoodbodies; #allbodiesaresexy). However, research has highlighted how sexualized (vs. non-sexualized) body-positive images increase women’s support of traditional beauty ideals and initiate other-objectification [16]. Thus, our results contribute to the current debates around body positivity (see [4] for a discussion). They suggest that the body-positivity phenomenon is complex and point to the need to understand the potential mechanisms underlying its effects. 

Indeed, exposure to both sexualized and non-sexualized body positivity was associated with downward social comparison, whereas exposure to sexualized beauty ideal content was linked to upward social comparison. Crucially, engagement in downward social comparison was shown to be a mechanism explaining the body-positivity impact on body satisfaction and positive mood, and PSNSU emerged as an important moderating variable in this pattern. Specifically, both body-positivity conditions’ effects on body satisfaction were mediated by downward social comparison, and the effects were bigger the higher participants’ PSNSU. Additionally, both sexualized and non-sexualized body-positivity conditions had an indirect effect on positive mood via engagement in downward social comparison, and the effect of the sexualized body-positivity condition was stronger the higher the PSNSU. Overall, these findings extend previous research emphasizing appearance social comparison as a key mechanism explaining SNS effects on young women’s body image [24,61,67,71,73,74]. Moreover, they extend previous research showing how PSNSU is a crucial individual difference to be considered in the context of body image research [47,71]. Importantly, the interpretation of these findings might raise some concerns. The goal of the body-positivity movement is to reject the narrow ideal of beauty by promoting the acceptance of all bodies, regardless of their shape, size, ethnicity, characteristics, and abilities [49]. However, scholars have pointed out that, despite its noble intentions, body positivity carries a certain degree of contradiction and ambiguity. Indeed, it continues to place emphasis on appearance and often perpetuates the representation of women as sexual objects (see [4] for a review). Our findings somewhat support this reasoning, showing that body-positivity messages can increase female viewers’ body satisfaction and positive mood because they engage in downward comparison. Such downward comparison is linked with a negative perception of non-conforming bodies (as bodies worse than their own), which defies the very positive idea behind the body-positivity movement. Such negative perception might be related to competition which, in Festinger’s theory [58], was deemed as one main underlying motivation for social comparison: the more important a certain domain is to individuals (e.g., physical appearance and beauty), the stronger the pressure toward uniformity and competition to gain it, the greater the readiness to allocate inferior and superior status to others, respectively, worse and better than them in that aspect. Thus, the beneficial effects of body positivity on body satisfaction and positive mood come at the expense of the potential negative perception of people with bodies not conforming to beauty standards. 

Young women’s self-objectification was not affected by the condition and these results were stable across platforms and medium (Instagram images and TikTok videos). Thus, extending Vendemia et al. [16], our findings suggest that body-positive posts have a similar impact on self-objectification as sexualized and idealized beauty content. This highlights once more the potential contradictions imbued in the movement, perpetuating a focus on appearance at the expense of other characteristics (Cohen et al., 2021). As in Guizzo et al. [10], the different conditions did not impact participants’ intentions to undergo cosmetic surgery. Interestingly, higher problematic SNS use (PSNSU) predicted higher self-objectification and cosmetic surgery intentions across studies, corroborating research showing a positive association between intense and problematic use of social networks and cosmetic surgery intentions [10] as well as self-objectification [31]. As discussed above PSNSU moderated the effects of conditions on body satisfaction. It seems that PSNSU might work differently on body image according to the type of content viewed. In fact, it appears that the benefits of exposure to body positivity (i.e., improved body satisfaction) are more powerful for those who are more prone to problematic SNS use who, arguably, are more likely to consider SNS an important source of body self-esteem [10,71]. Likewise, the negative impact of exposure to sexualized beauty ideals is stronger for those with high PSNSU, a result that supports existing literature on the role of social media use in social comparison [40,67,74].

### Limitations and Future Directions

Our research is not free of limitations. First, some of the effect sizes were small (i.e., direct effects of condition on body satisfaction, mood, and the degree of appearance comparison). They aligned with previous research on sexualized imagery [10] but were smaller compared to research on body positivity [14]. Therefore, despite being not negligible, any generalization should be taken with appropriate caution. 

Another important limitation of our studies is that we did not control participants’ levels of self-objectification and cosmetic surgery intentions pre-manipulation; therefore, we cannot test whether exposure to conditions actually reduces or increases women’s self-objectification and intentions to cosmetic surgery. Future research might address this gap using experimental designs including repeated measures of self-objectification and cosmetic surgery intentions. Furthermore, we did not consider whether the participants (or family members or friends) had previously undergone any type of cosmetic surgery, which might have further informed us about their interest in cosmetic surgery [95]. Moreover, in measuring intentions toward cosmetic surgery we did not differentiate among the existing types of cosmetic surgery, from the least (e.g., fillers) to the most invasive (e.g., breast augmentation), which would have provided us with additional information about actual intentions toward cosmetic surgery. 

Additionally, due to a procedural error, we did not measure participants’ body mass index, which would have been useful to ascertain that participants’ BMI distribution was not different across conditions; however, the randomization applied across conditions and the two studies should have helped overcome this issue. Moreover, the nature of our two studies (i.e., online questionnaires) raises the issue of the demand characteristics bias. Despite adopting some precautions, such as providing participants with a detailed cover story, participants often indicated at the end of the study to be aware of the real purpose of the study (64% in the Instagram study, 72% in the TikTok study). This may not be surprising considering that people are aware of the body-positivity movement’s intents. Still, demand characteristics might have biased our findings similarly to previous studies on this topic. Future research should implement strategies to better control such bias or use alternative measures 

Finally, another limitation may lie in how the mood has been measured. We used positive and negative emotions previously used (e.g., angry, happy). The only stable mood effects confirmed across platforms is the fact that sexualized beauty ideals have a detrimental effect on positive mood also in comparison with the sexualized body-positivity condition, while negative mood seems to decrease across all conditions compared to pre-exposure levels. These results partially contradict previous research [14,24] showing the beneficial effects of body positivity and the detrimental effects of fit inspiration on negative mood. Altogether the research on SNS impact on mood is quite inconsistent (see [10,77] for example of null findings) thus it is difficult to draw strong conclusions. Although similar in valence, the emotions we employed are different in nature and may vary in how they related to the stimuli or self (e.g., anger toward idealized body representation, anger towards one own body). Hence, it is difficult to understand what triggers each specific emotion. Future research might need to use more broad measures of mood (e.g., feeling good/bad) or focus on specific emotions and what they relate to.

Since our aim was to test the impact of exposure to SNS body positivity, we adopted an inclusive and ecological approach by selecting SNS content representing different types of bodies with various characteristics (e.g., disabilities; acneic skin; overweight bodies) in different poses and controlling just for the degree of sexualization of the stimuli. This approach does not allow us to draw conclusions on the effects of viewing specific types of body-positive messages, since we did not focus just on one type of body, size, ethnicity, and characteristic. Therefore, future research might focus on specific bodies and characteristics (e.g., body hair; disability) portrayed in body positivity to test their effects on young women’s body image. Importantly, our results showed that body positivity can increase young women’s body satisfaction and positive mood via engaging in downward comparison, which might be linked with competition and a negative perception of non-conforming bodies (as bodies worse than their own). Given the potential benefits and notable criticisms of the online body-positivity movement, future research should also investigate other mechanisms explaining sexualized and non-sexualized body-positivity effects on body image, such as other objectification [16], internalization of beauty ideals, and perception of women portrayed in body-positivity messages [16].

Future studies might also investigate potential reasons underlying differences across platforms. We found that the non-sexualized body-positivity condition led to higher appearance comparison when watching TikTok videos compared to static images on Instagram. As this is the first research analyzing body-positivity effects across the two platforms, the results should be taken with caution and are difficult to interpret. As discussed in Study 2′s discussion, specific features of the stimuli or the fact that we did not control for participants’ chronic appearance social comparison might explain this effect, but more studies are needed to draw any conclusion. 

## 6. Conclusions

Our work demonstrated for the first time that sexualization does not impair the expected benefits of exposure to SNS body-positive images/reels on young women’s body image. Importantly, we showed similar effects when considering static Instagram images and TikTok videos contributing to the very limited research on this latter platform. It also emphasized the key role of downward social comparison in explaining such effects and that individuals’ problematic SNS use is an individual difference worthy of consideration. Thus, our findings have important implications. They suggest that SNS body positivity, despite its beneficial effects on body image, might reinforce the emphasis on appearance and activate competitive behaviors, encouraging women to downward social comparison in order to enhance their body satisfaction. This novel insight should guide health professionals in their work with women on body concerns and interventions on body image. It can also make SNS developers and activists aware of the potential unwanted and counterproductive effects of such content on body image. Parents and institutions should be well informed about the benefits and risks associated with SNS exposure. Promoting educational campaigns to encourage safe and responsible SNS use among young women might be crucial for avoiding some of the most deleterious outcomes of SNS body content (e.g., sexualized beauty ideals). Finally, health professionals and researchers should continue to find strategies that enable individuals to build strong self-esteem and positive body image without engaging in social comparison and by nurturing more varied aspects of the self beyond physical appearance.

## Figures and Tables

**Table 1:** Demographic Information of Study 1 and Study 2.

		Study 1 (Instagram)	Study 2 (TikTok)
**Sexual Orientation % (*n*)**	Heterosexual	91% (324)	84.8% (268)
	Lesbian	0.8% (3)	2.5% (8)
	Bisexual	7% (25)	9.2% (29)
	Other/no answer	1.1% (4)	3.5% (11)
**Education % (*n*)**	Middle School	2.5% (9)	2.2% (7)
	High School	38.8% (138)	50.6% (160)
	BSc degree	32.9% (117)	25.0% (79)
	MSc degree	22.8% (81)	19.9% (63)
	Other	3.1% (11)	2.2% (7)
**Occupation % (*n*)**	Student	40.4% (144)	54.7% (173)
	Worker	35.4% (126)	25.3% (80)
	Working student	17.7% (63)	15.8% (50)
	Unemployed	6.5% (23)	4.1% (13)
**Age**	Range (19–32)	*M* = 24.98 *SD* = 3.69	*M* = 23.87 *SD* = 4.19
**Daily time spent on Social Networking Sites (SNS)**	Range (0–100%)	*M* = 34.17 *SD* = 15.53	*M* = 27.37 *SD* = 15.14

**Table 2:** Means and Standard Deviations of all the Dependent Variables across Conditions and Separated by Study.

		Non-Sexualized Body Positivity	Sexualized Body Positivity	Sexualized Beauty Ideals
		*M (SD*)	*M (SD*)	*M (SD*)
**Study 1** **Instagram**	1. Pre-study body satisfaction	45.65_a_ (29.40)	45.18_a_ (27.93)	44.09_a_ (28.19)
	2. Post-study body satisfaction	46.77_abc_ (30.04)	47.88_b_ (28.76)	37.72_c_ (29.93)
	3. Pre-study positive mood	47.22_a_ (22.05)	47.04_a_ (22.22)	45.05_a_ (22.05)
	4. Post-study positive mood	46.26_ab_ (23.79)	46.58_ab_ (23.44)	40.03_b_ (24.50)
	5. Pre-study negative mood	31.12_a_ (22.47)	36.43_a_ (21.98)	33.87_a_ (21.37)
	6. Post-study negative mood	28.69_b_ (23.12)	31.43_b_ (22.35)	31.22_b_ (24.46)
	7. Appearance comparison degree	2.47_a_ (2.60)	3.03_a_ (2.75)	4.47_b_ (3.09)
	8. Appearance comparison direction	0.55_a_ (0.68)	0.70_a_ (0.72)	−0.89_b_ (0.68)
	9. Self-objectification	−0.62_a_ (1.90)	−0.47_a_ (2.02)	−0.67_a_ (1.76)
	10. Cosmetic surgery intentions	4.46_a_ (1.64)	4.48_a_ (1.73)	4.32_a_ (1.78)
**Study 2** **TikTok**	1. Pre-study body satisfaction	47.19_a_ (27.11)	52.70_a_ (25.70)	44.87_a_ (27.01)
	2. Post-study body satisfaction	49.77_b_ (28.38)	57.47_b_ (24.83)	38.67_c_ (28.72)
	3. Pre-study positive mood	48.08_a_ (20.29)	51.10_a_ (24.00)	49.94_a_ (21.96)
	4. Post-study positive mood	48.91_ab_ (22.62)	52.78_a_ (24.76)	43.80_b_ (24.60)
	5. Pre-study negative mood	33.55_a_ (23.25)	30.69_a_ (21.85)	31.70_a_ (21.98)
	6. Post-study negative mood	28.23_b_ (22.51)	25.90_b_ (22.30)	31.14_ab_ (24.36)
	7. Appearance comparison degree	3.75_a_ (3.00)	2.38_b_ (2.48)	4.66_a_ (3.31)
	8. Appearance comparison direction	0.56_a_ (0.73)	0.72_a_ (0.64)	−0.90_b_ (0.79)
	9. Self-objectification	−0.90_a_ (1.69)	−1.16_a_ (1.94)	−0.92_a_ (2.01)
	10. Cosmetic surgery intentions	3.87_a_ (1.87)	3.71_a_ (1.75)	3.92_a_ (1.74)

Note: Means across each row (and columns for pre-post variables) that do not share the same subscript are significantly different from each other at *p* < 0.05 level (Bonferroni-adjusted).

**Table 3:** Zero-order Correlations among all the variables separated for Study 1 and Study 2.

		**1.**	**2.**	**3.**	**4.**	**5.**	**6.**	**7.**	**8.**	**9.**	**10.**
**Study 1-Instagram**	1. Pre-study body satisfaction	-									
	2. Post-study body satisfaction	0.91 **	-								
	3. Pre-study positive mood	0.45 **	0.45 **	*-*							
	4. Post-study positive mood	0.52 **	0.60 **	0.84 **	-						
	5. Pre-study negative mood	−0.16 **	−0.15 **	−0.46 **	−0.34**	*-*					
	6. Post-study negative mood	−0.19 **	−0.18 **	−0.43 **	−0.37 **	0.893 **	-				
	7. Appearance comparison degree	−0.25 **	−0.29 **	−0.21 **	−0.25 **	0.116 *	0.15 **	-			
	8. Appearance comparison direction	0.16 **	0.34 **	0.11	0.22 **	−0.05	−0.08	−0.29 **	-		
	9. Self-objectification	−0.20 **	−0.14 **	−0.18 **	−0.18 **	0.188 **	0.20 **	0.21 **	0.07	-	
	10. Cosmetic surgery intentions	−0.23 **	−0.21 **	−0.12 *	−0.13 *	0.1	0.14 **	0.17 **	0.08	0.26 **	-
		**1.**	**2.**	**3.**	**4.**	**5.**	**6.**	**7.**	**8.**	**9.**	**10.**
**Study 2-TikTok**	1. Pre-study body satisfaction	-									
	2. Post-study body satisfaction	0.88 **	-								
	3. Pre-study positive mood	0.54 **	0.52 **	-							
	4. Post-study positive mood	0.56 **	0.65 **	0.85 **	-						
	5. Pre-study negative mood	−0.14 *	−0.12 *	−0.36 **	−0.28 **	-					
	6. Post-study negative mood	−0.18 **	−0.19 *	−0.35 **	−0.32 **	0.873 **	-				
	7. Appearance comparison degree	−0.37 **	−0.39 **	−0.21 **	−0.27 **	0.216 **	0.25 **	-			
	8. Appearance comparison direction	0.28 **	0.46 **	0.19 **	0.33 **	−0.05	−0.15 *	−0.33 **	-		
	9. Self-objectification	−0.25 **	−0.23 **	−0.21 **	−0.20 **	0.227 **	0.23 **	0.36 **	−0.06	-	
	10. Cosmetic surgery intentions	−0.23 **	−0.18 **	−0.21 **	−0.15 **	0.252 **	0.25 **	0.17 **	−0.12	0.29 **	-

Note: * *p* < 0.05; ** *p* < 0.01.

**Table 4:** Descriptive Statistics (Means and Standard Deviations) of Joynt Analyses’ Dependent Variables Separated for Condition.

	Non-Sexualized Body Positivity	Sexualized Body Positivity	Sexualized Beauty Ideals
	*M (SD*)	*M (SD*)	*M (SD*)
1. Pre body satisfaction	46.37_a_ (28.29)	48.64_a_ (27.13)	44.46_a_ (27.57)
2. Post body satisfaction	48.17_b_ (29.25)	52.30_b_ (27.38)	38.18_c_ (29.29)
3. Pre positive mood	47.63_a_ (21.20)	48.91_a_ (23.09)	47.41_a_ (22.09)
4. Post positive mood	47.50_a_ (23.23)	49.43_a_ (24.20)	41.84_b_ (24.55)
5. Pre negative mood	32.79_a_ (22.79)	33.78_a_ (22.05)	32.82_a_ (21.64)
6. Post negative mood	28.47_b_ (22.78)	28.88_b_ (22.45)	31.18_b_ (24.35)
7. Appearance comparison degree	3.07_a_ (2.86)	2.73_a_ (2.64)	4.56_b_ (3.19)
8. Appearance comparison direction	0.56_a_ (0.70)	0.71_a_ (0.69)	−0.89_b_ (0.74)
9. Self-objectification	−0.75_a_ (1.81)	−0.79_a_ (2.00)	−0.79_a_ (1.89)
10. Cosmetic surgery intentions	4.18_a_ (1.77)	4.12_a_ (1.78)	4.13_a_ (1.77)

Note: Means across each row (and columns for pre-post variables) that do not share the same subscript are significantly different from each other at *p* < 0.05 level (Bonferroni-adjusted).

**Table 5:** Moderation model with Condition, PSNSU, and Their Two-Way Interactions as Predictors of Body Satisfaction (Post manipulation).

	*b*	*SE b*	*t*	*p*	*F(dfs)*	*R^2^*	ΔR^2^
**Model**				<0.001	816.88 (7664)	0.83	-
Intercept	−2.80	1.19	−2.35	0.019			
PSNSU	-0.27	0.17	−1.62	0.105			
X1	10.13	1.09	9.26	<0.001			
X2	8.29	1.2	6.9	<0.001			
Study	−1.59	0.93	−1.7	0.090			
Body satisfaction (Pre manipulation)	0.94	0.02	57.38	<0.001			
				0.006	5.13 (2664)	-	0.003
PSNSU × X1	0.63	0.24	2.64	0.009			
PSNSU × X2	0.83	0.32	2.62	0.009			

Note: Huber-White (HC0) heteroskedasticity-consistent standard error estimator was used to correct for homoskedasticities violations. X1 = Sexualized body positivity (=1) vs. sexualized beauty ideal condition (=0). X2 = Non-sexualized body positivity (=1) vs. sexualized beauty ideal condition (=0). PSNSU = problematic social networking sites use.

**Table 6:** Moderation model with Condition, PSNSU, and Their Two-Way Interactions as Predictors of the Direction of Social Comparison.

	*b*	*SE b*	*t*	*p*	*F(dfs)*	*R^2^*	ΔR^2^
**Model**				<0.001	118.78 (6529)	0.53	-
Intercept	−0.89	0.06	−14.25	<0.001			
PSNSU	−0.04	0.01	−2.82	0.005			
X1	1.6	0.07	21.67	<0.001			
X2	1.45	0.07	19.41	<0.001			
Study	0.01	0.06	0.11	0.912			
				0.006	5.11 (2, 529)	-	0.01
PSNSU x X1	0.05	0.02	3.1	0.002			
PSNSU x X2	0.04	0.02	2.32	0.021			

Note: Huber-White (HC0) heteroskedasticity-consistent standard error estimator was used to correct for homoskedasticities violations. X1 = Sexualized body positivity (= 1) vs. sexualized beauty ideal condition (= 0). X2 = Non-sexualized body positivity (= 1) vs. sexualized beauty ideal condition (= 0). PSNSU = problematic social networking sites use.

## Data Availability

Datasets are publicly archived in the Open Science Framework (OSF) at https://osf.io/jav7n/ (accessed on 28 December 2022).

## Supplementary Materials

The following supporting information can be downloaded at: https://www.mdpi.com/article/10.3390/ijerph20020991/s1, Stimuli pretests; Figure S1: PSNSU moderating effect on the relation between condition and post-exposure positive mood; Table S1: Manipulation checks’ descriptive statistics separated by study; Table S2: Moderation model with Condition, PSNSU, and Their Two-Way Interactions as Predictors of Positive Mood (Post- manipulation).
