# Supplementary material for: #SexyBodyPositive: When Sexualization Does Not Undermine Young Women’s Body Image
Source: Int J Environ Res Public Health. 2023 Jan 5;20(2):991. doi: 10.3390/ijerph20020991 (PMC9858851; doi:10.3390/ijerph20020991)
Supplement: Supplementary file 1 [file ijerph-20-00991-s001.zip › ijerph-2097936-supplementary.pdf]

Supplementary material

Stimuli pretest

The selection procedure of the stimuli was the same for Study 1 and Study 2. Instagram and TikTok contents were searched using hashtags related to the cultural beauty ideal (e.g., #beauty; #bikinigirl; #hot) and body positivity (e.g., #bopo; #bodypositive; #bodypositivity). All the stimuli were retrieved from public accounts on Instagram and TikTok and portrayed non-famous women who differed in terms of sexualization. For the sexualized beauty ideal condition of Study 1 (i.e., Instagram contents), we retrieved n = 10 images used in the study by Guizzo et. al (2021). For the remaining conditions (sexualized and non-sexualized body positivity in Study 1; sexualized beauty ideal, sexualized, and non-sexualized body positivity in Study 2), the stimuli were selected according to the following procedure which involved two stages.

1) As the aim of our project was to disambiguate the role of sexualization in body positivity contents compared to the cultural beauty ideal, the degree of sexualization of the images and videos retrieved portraying either body positivity or the cultural beauty ideal (Study 1 n = 46 images; Study 2 n = 57 videos) was evaluated through Hatton and Trautner's (2011) coding system by three female raters. Once reached the consensus, the raters assigned a sexualization-score (from 0 to 23) to each stimulus. According to Hatton and Trautner (2011), values from 0 to 4 represent non sexualized contents, scores between 5 and 9 indicate sexualized contents, and scores above 10 denote hypersexualized contents. Then, contents with the lowest scores on the sexualization index (Study 1: non-sexualized body positivity n = 14, M = 2.29, SD = 1.27; Study 2: non-sexualized body positivity n = 14, M = 2.29, SD = 1.59) and contents with the highest scores (Study 1: sexualized body positivity n = 14; M = 10.07; SD = 2.23; Study 2: sexualized body positivity n = 14, M = 11.07, SD = 2.52; beauty ideal n = 14, M = 12.79, SD = 2.22) were selected and subjected to a pretest.

2) We pretested an initial set of 28 images for Study 1 (14 sexualized body positivity contents and 14 non-sexualized body positivity contents) and 42 videos for Study 2 (14 beauty ideal-related contents, 14 sexualized body positivity contents, and 14 non-sexualized body positivity contents). For each photo and video, forty-three female participants aged 18 and 30 years old (M = 23.93; SD = 2.51) rated the degree of sexual objectification of the target (scale: 1 = "Not at all"; 5 = "Totally"), and how representative the content was of the body positivity movement (scale: 1 = "Not at all"; 7 = "Totally"). Definitions of sexualization and body positivity were provided along with each question. Hence, the stimuli ranked as the most representative of the three experimental conditions

were selected for the studies. Namely, participants perceived women promoting body positivity as body positive contents, regardless of the degree of sexualization, $F(3, 36) = 2.88$, $p = .05$. For the sexualized body positivity condition, we selected the body positive contents perceived as more sexualized (STUDY 1 $n = 10$, $M = 2.53$, $SD = .49$; STUDY 2 $n = 10$, $M = 2.48$, $SD = .20$), whereas for the non-sexualized body positivity condition, the less sexualized stimuli were chosen (STUDY 1 $n = 10$, $M = 1.19$, $SD = .10$; STUDY 2 $n = 10$, $M = 1.23$, $SD = .10$). Despite in the first stage of selection the degree of sexualization of women promoting body positivity appeared to be like that of women conforming to the beauty ideal for both studies ($ps > .284$), participants perceived women conforming to the beauty ideal more sexually objectified (STUDY 1 $n = 10$, M = 3.67, SD = .48; STUDY 2 $n = 10$, $M = 3.93$, $SD = .25$) compared to sexualized women promoting body positivity (STUDY 1 $p < .001$; STUDY 2 $p < .001$), as well as non-sexualized body positivity contents (STUDY 1 $p < .001$; STUDY 2 $p < .001$), $F(5,59) = 134.06$, p < .001, $\eta^2 = .92$. Additionally, women in the non-sexualized body positivity condition were less likely to be perceived sexually objectified compared to sexualized women promoting body positivity (STUDY 1 $p < .001$; STUDY 2 $p < .001$). For each type of condition (beauty ideal vs sexualized body positivity vs non-sexualized body positivity), the degree of sexualization did not differ between the two studies (all $p_s > .833$).

Finally, for both studies, in each experimental condition the stimuli ($n = 10$) were grouped together into one video made with PowerPoint (for Instagram contents) and VN - Video Editor (for TikTok contents). To enhance ecological validity, the transition between the stimuli in each video replicated the scrolling of Instagram images and TikTok videos on the main page of the respective social networks. Moreover, usernames, likes and comments were obscured while maintaining the interface of the two social networks. Contents related to sexualized beauty ideals depicted women conforming to the cultural beauty ideal in bikini and sensual poses; whereas body positivity contents portrayed women with bodies not conforming to the cultural beauty ideal either in bikini and sensual poses (sexualized body positivity condition) or clothed and in non-sexy poses (non-sexualized body positivity condition).

Manipulation checks analyses

We measured participants' perception of the images' sexualization and representativeness of the cultural beauty ideal. Responses were given on a 5-point Likert scale (1 = "Not at all", 5 = "Very much").

Study 1 – Instagram

A MANOVA was conducted on the beauty ideal item and sexualization item with condition (sexualized beauty ideal vs. sexualized body positivity vs. non-sexualized body positivity) as between-subjects factor. The condition effect was significant on both items (sexualization: $F(2, 353) = 121.83, p < .001, \eta_p^2 = .41$; beauty ideal: $F(2, 353) = 238.67, p < .001, \eta_p^2 = .57$). As shown in the table below, pairwise comparisons (Bonferroni corrected), showed that participants perceived the sexualized beauty ideal condition as the most sexualized compared to both body positivity condition. Importantly, the sexualized body positivity condition was perceived as significantly more sexualized than the non-sexualized body positivity condition. Moreover, the sexualized beauty ideal was perceived as portraying the beauty ideals (e.g., of thinness and perfection) significantly more than the other two body positivity conditions. Importantly, the two body positivity conditions did not differ in beauty ideals perception.

Study 2 – Tik Tok

The same analysis as above was conducted on the Tik Tok sample. The condition effect was significant on both sexualization ($F(2, 313) = 265.00, p < .001, \eta_p^2 = .63$) and beauty ideal ($F(2, 313) = 189.21, p < .001, \eta_p^2 = .55$) perceptions. Pairwise comparisons revealed a pattern of results identical to the Instagram images, see Table 1 below for descriptive statistics.

Table S1.

Manipulation checks' descriptive statistics separated by study

		**Non-sexualized body positivity**	**Sexualized body positivity**	**Sexualized beauty Ideals**
		M (SD)	*M (SD)*	*M (SD)*
**Study 1 Instagram**	1. Perceived sexualization	1.74_a (.79)	2.93_b (1.11)	3.59_c (.83)
	2. Perceived beauty ideals portrayal	2.15_a (.86)	1.96_a (.69)	4.06_b (.87)
**Study 2 TikTok**	1. Perceived sexualization	1.46_a (.65)	2.93_b (1.09)	4.21_c (.81)
	2. Perceived beauty ideals portrayal	2.11_a (.86)	1.90_a (.76)	4.02_b (.98)

Note: means with different subscripts across rows are statistically different one another at p < .05 (Bonferroni adjusted).

Additional moderation results on positive mood

Concerning positive mood, as shown in Table 2 below, a significant interaction between PSNSU and X1 was found but it did not significantly increase the amount of variance explained ($\Delta R^2 = .003$, F (2, 664) = 2.81, $p = .061$). Nevertheless, the higher participants' PSNSU the higher their positive mood in the sexualized body positivity condition ($b = .43$, $SE = .20$, $t = 2.16$, $p = .031$) compared to the sexualized beauty ideal

condition, which showed a reversed, although not significant, pattern of results (b = -.18, t = 1.02, p = .305) (see Figure 1). Positive mood in the non-sexualized body positivity condition was not significantly affected by PSNSU levels (b = .21, t = 1.06, p = .291)[1].

Table S2.

Moderation model with Condition, PSNSU, and Their Two-Way Interactions as Predictors of Positive Mood (Post- manipulation).

	b	*SE b*	*t*	*p*	*F(dfs)*	R^2
**Model**				<.001	330.41 (7,664)	.73
Intercept	-1.45	1.39	-1.04	.297		
PSNSU	-.19	.18	-1.03	.305		
X1	6.17	1.19	5.17	<.001		
X2	5.46	1.24	4.4	<.001		
Study	-1.32	.98	-1.35	.179		
Positive mood (Pre- manipulation)	.93	.02	41.91	<.001		
PSNSU x X1	.62	.27	2.33	.020		
PSNSU x X2	.40	.27	1.47	.143		

Note:

Huber-White (HC0) heteroskedasticity-consistent standard error estimator was used to correct for homoskedasticities violations.

X1 = Sexualized body positivity (= 1) vs. sexualized beauty ideal condition (= 0)

X2 = Non-sexualized body positivity (= 1) vs. sexualized beauty ideal condition (= 0)

PSNSU = problematic social networking sites use

[1] The same results were found when not controlling for pre-exposure positive mood, but the model explained a significant lower amount of variance, $F(6, 665) = 11.16, p < .001, R^2 = .09$.

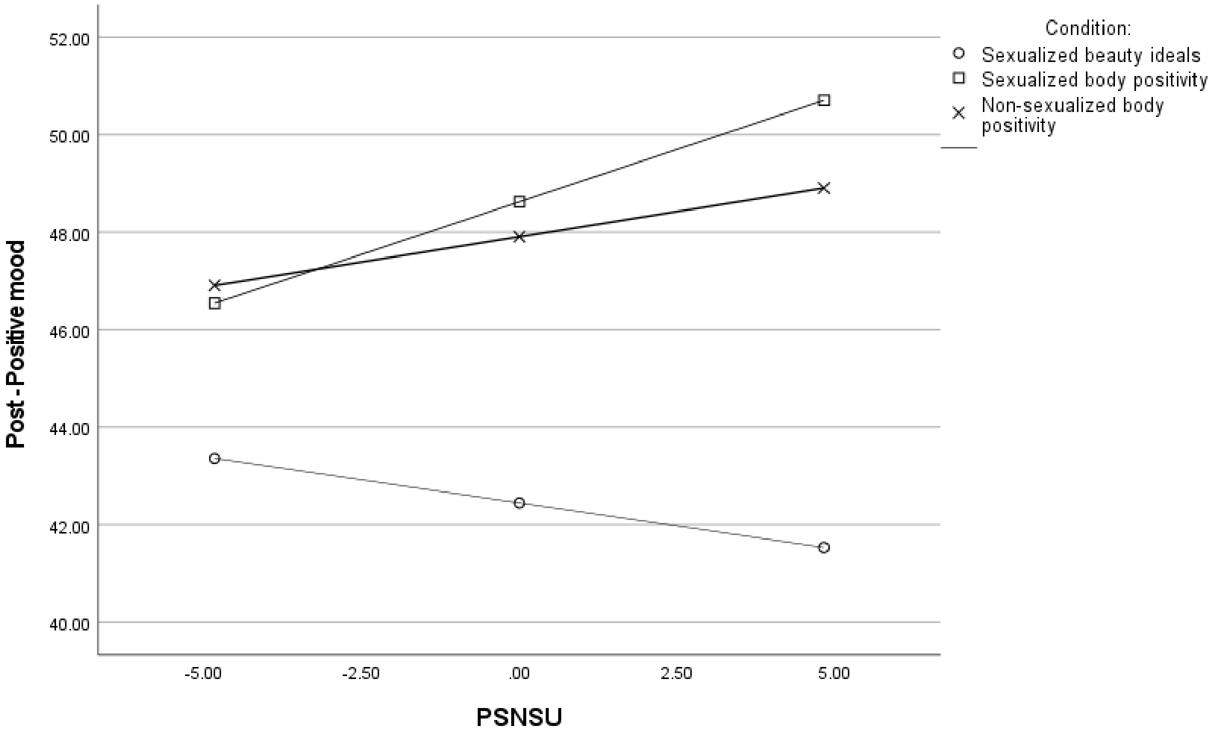

Figure S1. *PSNSU moderating effect on the relation between condition and post-exposure positive mood.*
